# Acute Esophageal Necrosis Secondary to Gastric Volvulus Presenting With Massive Aspiration of Upper Gastrointestinal Contents: A Case Report

**DOI:** 10.7759/cureus.103618

**Published:** 2026-02-14

**Authors:** Hatem Ahmed, Rouaa Chikho, Muhammad B Jamshaid, Motaz Almahmood, Roy Arslan Ahmed

**Affiliations:** 1 Internal Medicine, Phoenixville Hospital/Tower Health Medical Group, Phoenixville, USA

**Keywords:** acute esophageal necrosis (aen), black esophagus, nosocomial aspiration pneumonia, organo-axial gastric volvulus, paraesophageal hernia, upper gastrointestinal(ugi) bleeding

## Abstract

Acute esophageal necrosis (AEN), or *black esophagus*, is a rare endoscopic syndrome typically encountered in critically ill patients. AEN associated with gastric volvulus is particularly uncommon and may be underrecognized when volvulus appears clinically asymptomatic. An 87-year-old woman admitted for pelvic fractures was incidentally found on CT to have a large paraesophageal hernia with organoaxial gastric volvulus and suspected gastric outlet obstruction, without gastrointestinal symptoms. She developed acute hypoxemic respiratory failure; during emergent intubation, a large volume of upper gastrointestinal contents (coffee-ground material) refluxed into the airway, consistent with massive aspiration. Bedside esophagogastroduodenoscopy relieved a functional obstruction in the upper third of the esophagus consistent with volvulus reduction and revealed extensive circumferential AEN with proximal extension. Despite supportive management, she progressed to septic shock with multiorgan failure and died after transition to comfort-focused care. This case highlights that paraesophageal hernia-associated organoaxial gastric volvulus can deteriorate abruptly with life-threatening aspiration despite an initially absent gastrointestinal symptom burden, underscoring the need for early recognition and close monitoring.

## Introduction

Acute esophageal necrosis (AEN), or *black esophagus*, is a rare and ominous syndrome defined endoscopically by diffuse, circumferential black discoloration of the esophageal mucosa with an abrupt transition at the gastroesophageal junction and variable proximal extension [1]. AEN is reported in approximately 0.01%-0.28% of esophagogastroduodenoscopies [1], and it carries a grave prognosis because it typically arises in the setting of profound physiologic stress and critical illness. Short-term mortality approaches one-third of cases, reflecting the severity of the underlying precipitant rather than mucosal injury alone [2-3].

AEN is commonly explained by a *two-hit* mechanism in which impaired esophageal perfusion, particularly affecting the classically vulnerable distal esophagus, is compounded by chemical injury from refluxed gastric contents and reduced mucosal defenses in debilitated patients [1]. Mechanical foregut derangements such as gastric volvulus secondary to large hiatal hernias can intensify both insults through acute obstruction with retained gastric contents and regional perfusion compromise [1].

We report a rare presentation of a lethal intersection of foregut obstruction and ischemic injury: an incidentally identified paraesophageal hernia with organoaxial gastric volvulus complicated by extensive AEN with proximal extension and massive coffee-ground aspiration. This case is distinctive for its aspiration-led, rapidly fatal presentation despite the absence of initial gastrointestinal symptoms.

## Case presentation

**Figure 1:**
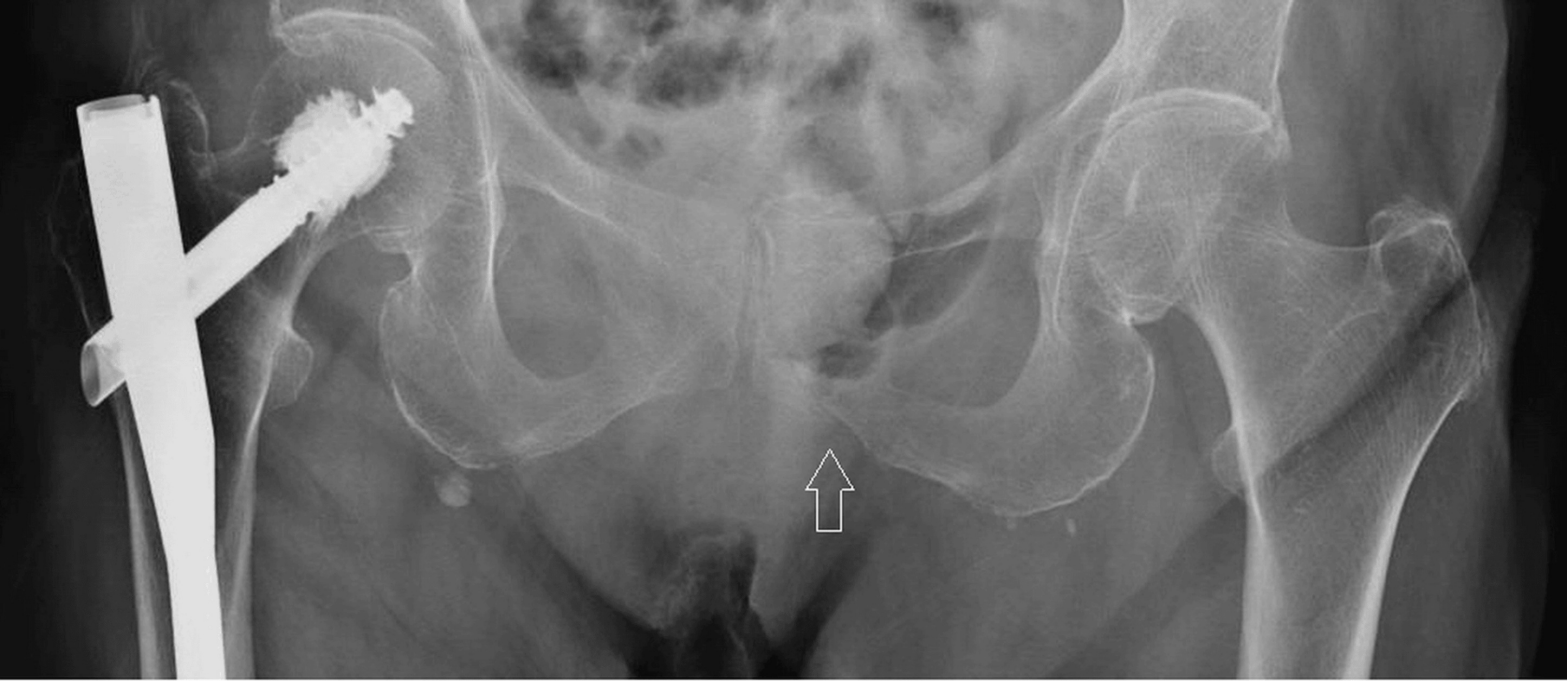
Pelvic radiograph demonstrating the left inferior pubic ramus fracture. Anteroposterior pelvic radiograph shows subtle cortical disruption of the left inferior pubic ramus (arrow), suspicious for a nondisplaced fracture. Prior fixation hardware in the right proximal femur is also visualized.

An 87-year-old female with a history of large hiatal hernia and chronic iron-deficiency anemia (baseline hemoglobin approximately 10 g/dL - reference range 12.0-15.5 g/dL) presented to the emergency department (ED) with worsening sacral pain and functional decline after a mechanical fall five days earlier. Pelvic radiographs demonstrated a left inferior pubic ramus fracture (Figure 1). The patient was hemodynamically stable on presentation and denied any complaints except for sacral pain.

**Figure 2:**
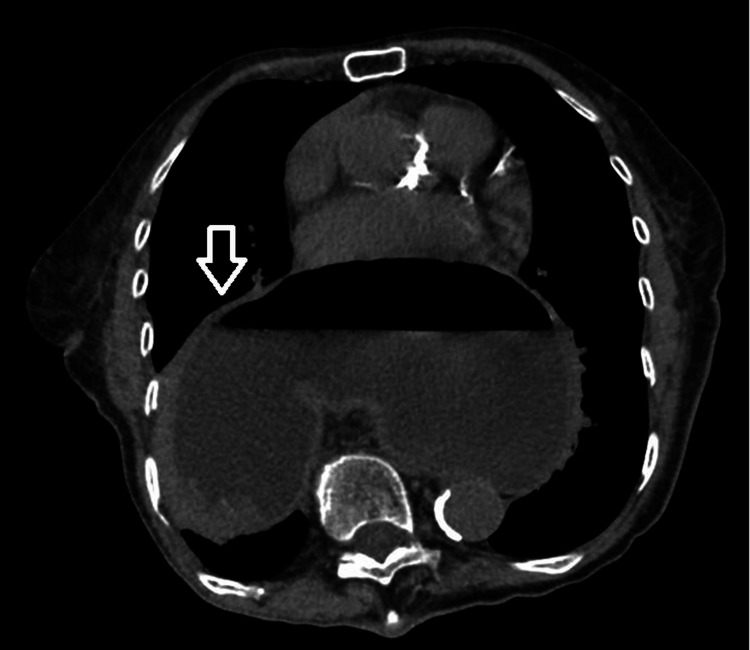
CT evidence of paraesophageal hernia with organoaxial gastric volvulus (arrow). Computed tomography (CT) of the abdomen and pelvis demonstrates a large hiatal (paraesophageal) hernia containing the stomach, with organoaxial gastric volvulus (the greater curvature positioned superior to the lesser curvature). The stomach is distended with an air-fluid level, raising concern for partial gastric outlet obstruction.

On presentation, she was hemodynamically stable. Physical examination was notable primarily for tenderness over the sacrum/pelvic girdle. Computed tomography (CT) of the abdomen and pelvis confirmed bilateral sacral fractures. CT also demonstrated a large paraesophageal hernia with organoaxial gastric volvulus and concern for gastric outlet obstruction (Figure 2). The patient denied gastrointestinal symptoms (no nausea, vomiting, abdominal pain, hematemesis, dysphagia, or reflux-related complaints). General surgery was consulted and recommended conservative management/observation given her lack of gastrointestinal symptoms. Because she had no clinical features of acute obstruction at that time, nasogastric decompression was not pursued.

**Figure 3:**
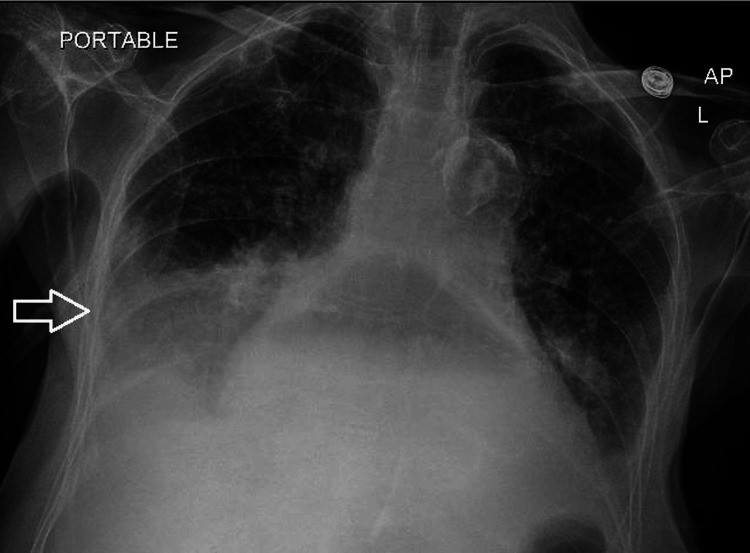
Portable chest radiograph demonstrating right basilar consolidation. Portable anteroposterior (AP) chest radiograph demonstrates right lung base consolidation (arrow), consistent with atelectasis and/or pneumonia. Small bilateral pleural effusions are present. The study also shows cardiomegaly and a large hiatal hernia containing stomach with a visible air-fluid level, consistent with intrathoracic herniation of gastric contents.

On hospital day 1, the patient developed acute hypoxemia with wheezing (SpO₂ 83% on room air) that improved with supplemental oxygen (2 L via nasal cannula) and nebulization. Chest radiography suggested right lower lobe atelectasis versus early pneumonia (Figure 3), and she reported dark, loose stools concerning for melena (hemoglobin 9.2 g/dL- reference range 12.0-15.5 g/dL). Within 24 hours, she abruptly deteriorated with respiratory distress and altered mental status, and repeat chest radiography demonstrated a worsening large right-sided consolidation. During emergent endotracheal intubation, a large volume of dark, coffee-ground material was observed refluxing into the airway, consistent with massive aspiration of upper gastrointestinal contents; an orogastric tube drained a large quantity of similar coffee-ground material.

The patient was transferred to the intensive care unit (ICU), where her hemoglobin decreased to 7.0 g/dL (from 9.2 g/dL the prior day), prompting transfusion of one unit of packed red blood cells. Intravenous proton pump inhibitor (PPI) therapy was initiated, and gastroenterology was consulted for suspected upper gastrointestinal bleeding. Further laboratory studies are summarized in Table 1.

**Table 1:** Laboratory results on ICU presentation.

Laboratory test	Result	Reference range
Hemoglobin (g/dL)	7.0	12.0-15.5
White blood cell count (×10⁹/L)	20.3	4.0-11.0
Platelets (×10⁹/L)	562	150-450
Blood urea nitrogen (mg/dL)	33	7-20
Creatinine (mg/dL)	0.74	0.6-1.1
Lactate (mmol/L)	3.4	0.5-2.2
INR	1.3	0.8-1.2

**Figure 4:**
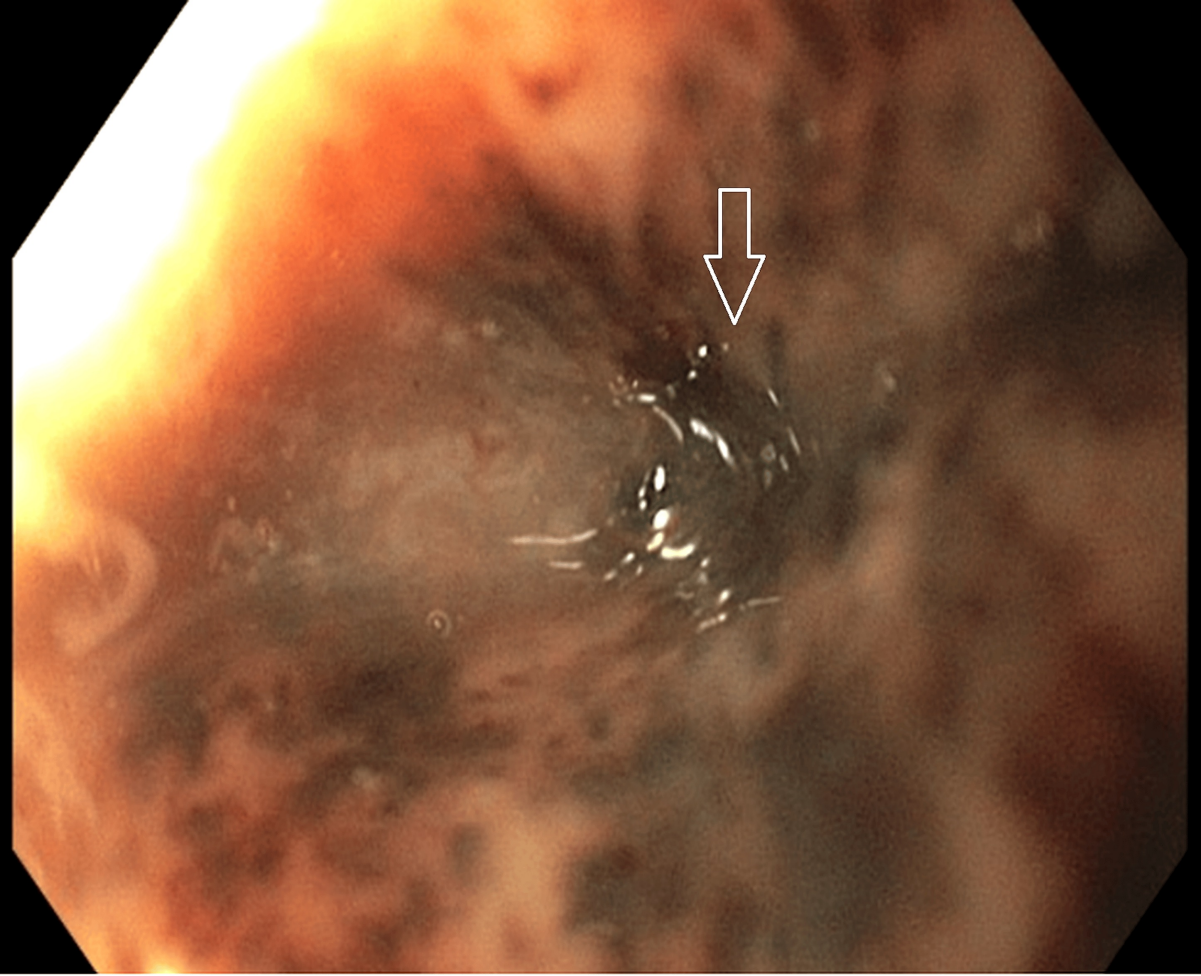
Proximal esophageal functional obstruction relieved during bedside EGD. Bedside esophagogastroduodenoscopy (EGD) shows significant narrowing/functional obstruction in the upper third of the esophagus (arrow). Gentle endoscopic advancement relieved resistance with subsequent opening of the lumen, consistent with endoscopic reduction of volvulus-related obstruction.

**Figure 5:**
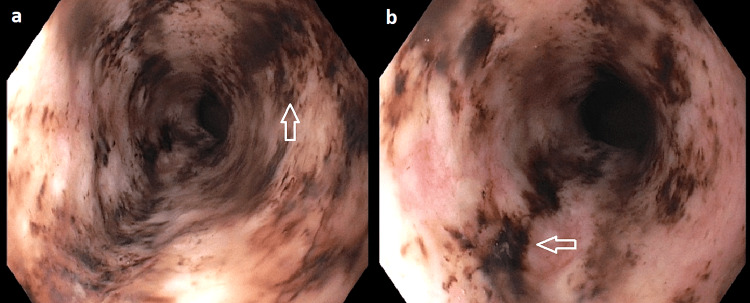
Acute esophageal necrosis with proximal extension. (a-b) Endoscopic images demonstrate diffuse, circumferential black discoloration of the esophageal mucosa consistent with acute esophageal necrosis (*black esophagus*) (arrow), with proximal extension to the upper third of the esophagus.

**Figure 6:**
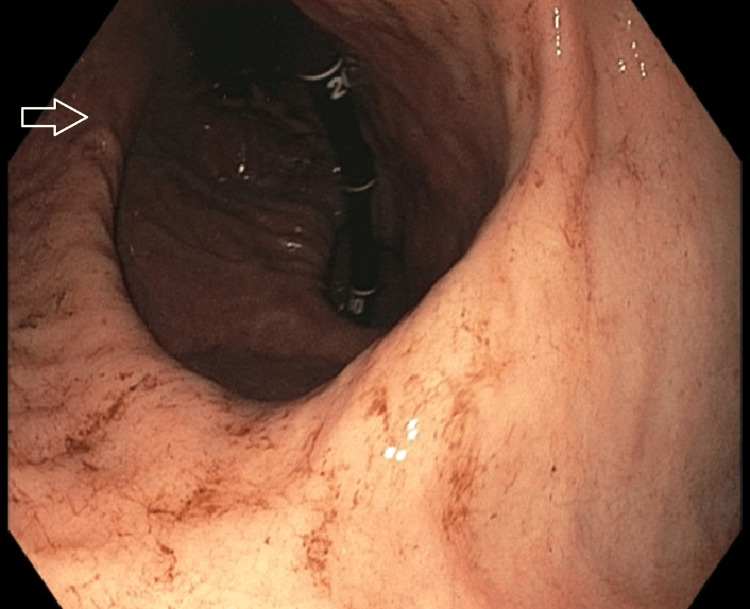
Paraesophageal hernia with distorted gastric anatomy and mucosal erosions. Endoscopic view of the stomach shows a large paraesophageal hernia with distorted gastric anatomy and scattered erosions within the hernia sac (arrow), as visualized during bedside upper endoscopy.

Bedside esophagogastroduodenoscopy (EGD) was performed and demonstrated a functional obstruction encountered in the upper third of the esophagus (Figure 4). Gentle passage of the endoscope relieved the resistance, consistent with reduction of volvulus-related obstruction. Diffuse, circumferential black discoloration of the esophageal mucosa consistent with AEN, extending from the distal esophagus proximally to the proximal/upper third (Figure 5). A large paraesophageal hernia with distorted gastric anatomy and scattered erosions (Figure 6), and two clean-based duodenal bulb ulcers without stigmata of active bleeding.

The patient remained intubated for acute hypoxemic respiratory failure and was treated for aspiration pneumonia with broad-spectrum antibiotics and supportive care. Despite vasopressor support and continued medical therapy (including intravenous PPI), she developed progressive septic shock with acute kidney injury, metabolic acidosis, and ongoing melena. Surgical intervention for the paraesophageal hernia/volvulus was not pursued due to her critical illness and poor physiologic reserve. After discussion of prognosis and lack of feasible operative options, her family elected comfort-focused care. The patient passed away within 24 hours of transition to palliative management.

## Discussion

AEN, or *black esophagus*, is an uncommon but ominous syndrome characterized endoscopically by diffuse circumferential black discoloration of the esophageal mucosa with a sharp transition at the gastroesophageal junction and variable proximal extension [1]. Clinically, AEN most often occurs in debilitated patients under substantial physiologic stress and is associated with high morbidity and mortality that largely reflect the severity of the underlying precipitating illness rather than mucosal injury alone [1].

AEN is generally understood to arise from combined ischemic injury and mucosal insult from refluxed gastric contents in a susceptible host (*two-hit* physiology) [1]. In patients with foregut mechanical pathology, such as gastric volvulus, obstruction, and gastric stasis, may amplify reflux burden may be amplified, while compromised perfusion increases ischemic vulnerability, creating conditions that can precipitate extensive mucosal necrosis [1,4]. In the specific setting of gastric volvulus, a recent comprehensive review identified only six published cases of co-occurring volvulus and AEN and proposed that volvulus contributes to AEN by limiting tissue perfusion and promoting massive reflux of retained gastric contents on compromised esophageal mucosa [4].

In our patient, CT demonstrated a large paraesophageal hernia with organoaxial gastric volvulus and suspected gastric outlet obstruction, creating a plausible anatomic substrate for retained gastric contents, retrograde reflux, and ischemic vulnerability. The bedside EGD was clinically notable for encountering functional obstruction in the upper third of the esophagus that was relieved with gentle passage, consistent with volvulus reduction followed by identification of extensive circumferential esophageal necrosis.

AEN classically predominates in the distal esophagus, and proximal-only involvement is rare. In a systematic review, distal disease accounted for 51% of cases, pan-esophageal for 36%, and proximal-only disease for 2% [5]. Most AEN cases present with upper gastrointestinal bleeding (hematemesis, coffee-ground emesis, melena), prompting endoscopic evaluation [5-6]. A distinctive feature of the present case was that the clinical course was *respiratory-declining*, with massive aspiration of coffee-ground material during emergent airway management preceding definitive endoscopic confirmation of AEN. This pattern emphasizes that in paraesophageal hernia with organoaxial rotation and suspected obstruction, life-threatening aspiration and rapid respiratory deterioration may precede or overshadow classic gastrointestinal symptoms.

Guidance for AEN management remains largely supportive: resuscitation and treatment of the precipitating illness, nil per os/bowel rest, and high-dose intravenous PPI [1,5]. In a systematic review, intravenous PPI therapy was used in the majority of reported cases (84%), and surgery was uncommon (4%), typically reserved for complications such as perforation [5]. In volvulus-associated AEN, reported management similarly centers on aggressive supportive care while addressing the precipitating mechanical process when feasible, with the choice of endoscopic versus operative intervention driven by clinical stability and concern for ischemia or perforation [4-5]. In our patient, initial management was conservative because the volvulus was identified in the setting of clinical stability and absent gastrointestinal symptoms; however, she subsequently deteriorated precipitously with aspiration and shock, at which point definitive surgical correction was no longer pursued due to critical illness and limited physiologic reserve. This trajectory highlights the narrow window in which *asymptomatic* volvulus may become unstable and catastrophic.

The larger clinical decision point raised by this case concerns the management of a paraesophageal hernia with volvulus that is labeled *asymptomatic*. The 2024 Society of American Gastrointestinal and Endoscopic Surgeons (SAGES) guideline notes that an *asymptomatic* paraesophageal hernia requires careful reassessment because many patients have non-gastrointestinal symptoms (e.g., dyspnea, exercise intolerance, anemia) that may be attributable to the hernia; the guideline also highlights micro-aspiration as a potential sequela and explicitly states that the panel considers pre-existing organoaxial rotation a concerning feature [7]. Although shared decision-making and patient frailty are central to management, this guidance supports heightened vigilance when organoaxial rotation is present, particularly when imaging suggests obstruction or when early respiratory changes emerge. Accordingly, the combination of organoaxial rotation and radiographic concern for obstruction should prompt early consideration of aspiration risk and close monitoring even when initial gastrointestinal symptoms are minimal or absent.

## Conclusions

This case adds to the limited literature describing AEN in the setting of gastric volvulus and highlights an especially catastrophic phenotype in which a radiographically apparent organoaxial volvulus/paraesophageal hernia initially considered clinically silent progressed rapidly to extensive AEN and massive aspiration with fatal multiorgan failure. Clinically, this report reinforces that paraesophageal hernia with organoaxial rotation (particularly when imaging suggests obstruction) should prompt heightened vigilance for aspiration and sudden respiratory deterioration, even when gastrointestinal symptoms are absent. Early recognition of aspiration risk and foregut obstruction may represent actionable targets to guide closer monitoring, escalation of evaluation, and timely multidisciplinary reassessment to prevent catastrophic outcomes. In line with recent guidance emphasizing reassessment of “asymptomatic” paraesophageal hernia and the recognized risk of micro-aspiration, clinicians should maintain a high index of suspicion for aspiration and ischemic complications when organoaxial rotation or obstruction is identified, even in the absence of initial gastrointestinal complaints.
